# Supplementary material for: Altered Pharmacokinetics of Ropivacaine in Patients Undergoing Laparoscopic Major Hepatectomy
Source: Pharmaceutics. 2025 Mar 18;17(3):386. doi: 10.3390/pharmaceutics17030386 (PMC11944703; doi:10.3390/pharmaceutics17030386)
Supplement: Supplementary file 1 [file pharmaceutics-17-00386-s001.zip › pharmaceutics-3487989-supplementary.pdf]

Supplementary Table S1. Total Plasma Ropivacaine Concentrations (ng·mL^{-1})

Time (h)	1	2	3	4	5	6	7	8	9	10	Mean (SD)
0.25	935.3	2094.7	3215.4	1359.3	1061.4	1092.9	1635.0	1816.0	3320.7	1287.9	1781.9 (861.3)
0.5	741.9	2129.9	3825.0	1259.1	1558.0	2138.5	1513.8	1825.1	3570.9	1485.2	2004.7 (982.9)
0.75	819.6	2178.5	3348.0	1354.0	1761.8	2181.3	1440.0	2157.8	3597.9	1476.4	2031.5 (876.0)
1	1055.0	1985.3	3590.6	1541.7	1557.5	2457.2	1244.9	1543.7	2894.7	1404.1	1927.5 (811.1)
1.5	815.0	1280.5	2770.5	2034.0	1164.4	2177.2	1061.0	1931.0	2414.2	1338.4	1698.6 (652.7)
2	629.6	1511.9	2947.3	1461.7	1080.5	2015.2	1100.9	1921.8	2375.7	1411.1	1645.6 (682.8)
4	254.9	1656.2	1935.1	1996.9	845.7	2057.7	698.9	1194.4	1809.0	1045.9	1349.5 (629.0)
24	294.2	154.6	583.9	656.9	382.0	859.0	69.5	312.0	200.7	299.9	381.3 (246.2)

Notes: Data are presented as means (SD).

Supplementary Table S2. Free Plasma Ropivacaine Concentrations (ng·mL^{-1})

Time (h)	1	2	3	4	5	6	7	8	9	10	Mean (SD)
0.25	93.3	80.1	116.4	52.9	99.4	36.1	111.8	78.8	90.8	82.2	84.2 (24.8)
0.5	59.5	73.8	74.4	65.0	71.5	65.0	98.5	47.9	108.3	82.8	74.7 (18.0)
0.75	58.2	48.6	61.7	71.0	83.6	73.2	79.7	52.5	88.5	100.9	71.8 (16.8)
1	46.7	42.7	60.9	108.0	113.2	114.5	102.5	99.6	145.6	133.6	96.7 (35.3)
1.5	69.3	72.5	96.6	168.4	108.3	123.8	88.9	113.1	132.9	141.5	111.5 (31.3)
2	60.7	65.1	91.4	155.3	99.6	116.1	82.9	109.3	118.6	137.0	103.6 (30.1)
4	19.1	34.7	47.4	55.5	61.9	75.6	31.5	70.6	78.3	88.8	56.3 (22.9)
24	9.95	3.15	13.5	26.4	11.9	27.9	1.35	9.07	5.96	27.1	13.6 (10.1)

Notes: Data are presented as means (SD).

Supplementary Table S3. Free Ropivacaine Fraction (%)

Time (h)	1	2	3	4	5	6	7	8	9	10	Mean (SD)
0.25	10.0	3.8	3.6	3.9	9.4	3.3	6.8	4.3	2.7	6.4	5.4 (2.6)
0.5	8.0	3.5	1.9	5.2	4.6	3.0	6.5	2.6	3.0	5.6	4.4 (1.9)
0.75	7.1	2.2	1.8	5.2	4.7	3.4	5.5	2.4	2.5	6.8	4.2 (2.0)
1	4.4	2.2	1.7	7.0	7.3	4.7	8.2	6.5	5.0	9.5	5.6 (2.5)
1.5	8.5	5.7	3.5	8.3	9.3	5.7	8.4	5.9	5.5	10.6	7.1 (2.2)
2	9.6	4.3	3.1	10.6	9.2	5.8	7.5	5.7	5.0	9.7	7.1 (2.6)
4	7.5	2.1	2.4	2.8	7.3	3.7	4.5	5.9	4.3	8.5	4.9 (2.3)
24	3.4	2.0	2.3	4.0	3.1	3.2	1.9	2.9	3.0	9.0	3.5 (2.1)

Notes: Data are presented as means (SD).

Supplementary Table S4. Plasma AAG Concentration ($\mu g \cdot mL^{-1}$)

Time (h)	1	2	3	4	5	6	7	8	9	10	Mean (SD)
0	833.0	1961.9	2078.6	1496.0	1455.9	1599.9	1406.0	1851.7	1679.3	835.1	1519.7 (422.6)
0.25	1539.9	2381.4	2685.6	1831.9	1602.6	2099.2	1557.0	2236.2	2172.4	1159.2	1926.5 (466.4)
0.5	1257.9	2875.9	2437.2	1717.6	1811.3	2061.9	2497.5	1950.3	2315.1	1577.9	2050.2 (486.5)
0.75	1513.9	2523.1	2767.9	1794.9	1891.5	1891.1	2393.6	2209.8	2413.4	1288.9	2068.8 (470.6)
1	1978.6	2337.3	2449.1	2056.8	1922.3	2048.5	1398.9	2650.5	2262.8	1224.3	2032.9 (443.7)
1.5	1489.7	2270.7	2620.7	1824.4	2586.0	2055.2	1627.3	2495.3	2170.6	971.2	2011.1 (534.0)
2	1815.9	2236.2	2507.7	1734.5	1840.7	2281.3	1477.1	1609.4	2184.8	1105.7	1879.3 (426.4)
4	1271.8	2637.1	2563.6	1953.8	1535.9	2055.2	1919.0	1785.5	2195.5	791.4	1870.8 (563.6)
24	2295.9	3057.3	2401.6	1971.4	2286.2	1734.5	2719.0	2420.1	2197.3	1453.1	2253.6 (460.4)

Notes: Data are presented as means (SD).
